# Is erythropoietin a worthy candidate for traumatic brain injury or are we heading the wrong way?

**DOI:** 10.12688/f1000research.8723.1

**Published:** 2016-05-19

**Authors:** Giovanni Grasso, Concetta Alafaci, Pietro Ghezzi

**Affiliations:** 1 Section of Neurosurgery, Department of Experimental Biomedicine and Clinical Neurosciences (BIONEC), University of Palermo, Palermo, 90100, Italy; 2 Department of Neurosurgery, University of Messina, Messina, 98100, Italy; 3 Brighton and Sussex Medical School, Falmer, BN1 9PX, UK

**Keywords:** Traumatic brain injury, Neuroprotection, Erythropoietin

## Abstract

Traumatic brain injury (TBI) is a leading cause of death and disability in the modern society. Although primary prevention is the only strategy that can counteract the primary brain damage, numerous preclinical studies have been accumulated in order to find therapeutic strategies against the secondary damage. In this scenario erythropoietin (EPO) has been shown to be a promising candidate as neuroprotective agent. A recent clinical trial, however, has shown that EPO has not an overall effect on outcomes following TBI thus renewing old concerns.  However, the results of a prespecified sensitivity analysis indicate that the effect of EPO on mortality remains still unclear.

In the light of these observations, further investigations are needed to resolve doubts on EPO effectiveness in order to provide a more solid base for tailoring conclusive clinical trials.

Traumatic brain injury (TBI) is one of the major causes of death and disability in our society ^1^. TBI can provides heterogeneous effects since, in addition to the primary injury, it is associated with the so-called secondary brain injury where inflammation, excitotoxicity, ischemia, edema participate in worsening the clinical scenario ^13^. Several pre-clinical studies have been conducted in order to identify neuroprotective agents able to counteract the secondary tissue damage and improve clinical outcomes ^8^. However, translation to the clinical trials has been discouraging and the treatment of TBI remains great challenge worldwide.

In both pre-clinical and clinical studies, erythropoietin (EPO) has been recognized for nearly two decades as a potent neuroprotective agent with a multifaceted, hematopoiesis-independent action profile ^4^. The discovery that EPO has neuroprotective functions apart from regulating erythropoiesis ^3^ was unexpected and prompted numerous studies showing a protecting role through antiapoptotic, antioxidative and anti-inflammatory, angiogenic and neurotrophic mechanisms ^7, 9^.

The recent conclusion of the EPO-TBI, double-blind randomized controlled trial ^10^, has renewed old concerns. This clinical study was undertaken in 29 centers in seven countries. A total of 606 patients were randomly selected. EPO was given to 308 patients in a dose of 40,000 units subcutaneously, while 298 patients received a placebo, consisting of 0.9% sodium chloride,. Both EPO and placebo were administered once per week for a maximum of three doses. Randomization was stratified by severity of traumatic brain injury (moderate *vs.* severe) and participating site ^10^. The primary outcome, consisting of improvement in the patients’ neurological status was measured at 6 months follow-up. It was summarized as a binary midpoint reduction of their extended Glasgow Outcome Scale (GOS-E) level, which was defined as a GOS-E of 1–4 (death, vegetative state, and severe disability) or a GOS-E of 5–8 (moderate disability and good recovery). In addition, mortality, proximal deep venous thrombosis and occurrence of general thrombotic events were assessed as secondary outcomes measures ^10^.

The authors found that EPO did not reduce the number of patients with a GOS-E level of 4 or lower, and did not affect the incidence of deep venous thrombosis events.

Overall, the results of this international multicenter randomized placebo-controlled trial suggest that EPO may not be useful in TBI. This result is in contrast with a number of experimental studies suggesting that EPO might improve neurological outcomes following TBI. However, the results of a prespecified sensitivity analysis adjusting for covariates indicate that the effect of EPO on mortality remains to be better investigated. Notably, although in this study EPO did not have an overall effect on survival ^10^, when adjusted for illness severity according to the IMPACT-TBI predicted probability of a poor outcome, 6-months mortality was lower in patients given EPO than in those who received placebo.

Although the authors suggest caution in the interpretation of these mortality findings, we believe this question is worthy of note and remains to be addressed. The time window for EPO administration following TBI and its dose regimen are the main arguments. In this study a 24 hours time window and a dose of 40,000 units was chosen. It must be taken into account that earlier preclinical studies showed that recombinant human EPO treatment at a dose of 1000 IU/kg administered every 8 hours starting following TBI, is effective as neuroprotective agent ^5^. The dose used in the study by Nichol and collaborators ^14^ is the lowest dosage known to be effective in the experimental settings, and the time for the first administration, an average of 18.6 hours after TBI, would initiate a neuroprotective program in a late secondary damage. The small dose used, time and frequency of administration could contribute to the unfavorable results from this clinical trial. Neuroprotective drugs should be administered as soon as possible and as long as the pathological cascades occur. EPO dose and therapeutic duration were clearly dictated by the concerns on the safety of recombinant human EPO. It is well known that all the information available regarding the safety of EPO comes from its non-neurologic use ^15^. Using the information accumulated on EPO safety in patients affected by chronic anemia and put into practice for the management of TBI can be dangerous since the interaction between EPO and various physiologic variables, in addition to drugs commonly used in TBI patients, are unknown.

Additionally, besides its fame of a well-tolerated drug, recent reports of adverse effects associated with the chronic administration of recombinant EPO (i.e. hypertension, hypertensive encephalopathy, seizures, and thrombotic/vascular events) have raised new concerns ^6^. Although in experimental and clinical studies, including this randomized trial, no adverse effects during EPO treatment were observed, it is unknown what the effect in patients with a raised hemoglobin concentration would be.

Taken collectively, the findings of this recent clinical trial ^11^, together with those from previous randomized studies ^2, 12, 14^, suggest that EPO might decrease mortality in this patient group.

The overall disappointing results of the clinical trials reported over the time could be due to protocol and dosage problems, and one should also bear in mind that it is not always possible to translate animal research to the clinical scenario, which is more complex and less controlled.

More attention should be paid in conducting clinical trials in order to obtain sufficient information regarding therapeutic time window, dosage, duration of therapy and safety. The uncertain results so far obtained put EPO at risk of being discarded as thoroughly as it was initially welcomed as a miracle drug. Meanwhile, better information on the spectrum of biological actions of EPO and the underlying mechanisms would provide a more solid base for tailoring conclusive clinical trials.

In the light of these observations, further investigations are required to resolve such uncertainties especially when issues as optimal dosages, therapeutic time window, and duration of therapy deserve to be clarified.
