# Supplementary material for: Is erythropoietin a worthy candidate for traumatic brain injury or are we heading the wrong way?
Source: F1000Res. 2016 May 19;5:911. [Version 1] doi: 10.12688/f1000research.8723.1 (PMC4879931; doi:10.12688/f1000research.8723.1)
Supplement: Peer review history

# Peer review: Referee response for version 1

**DOI:** 10.5256/f1000research.9385.r13936

**Published:** 2016-05-24

**Authors:** Alessandro Landi

**Affiliations:** 1 Department of Neurology and Psychiatry, Division of Neurosurgery, Policlinico Umberto I, Sapienza University of Rome, Rome, Italy

The title and abstract are exhaustive and clear, appropriate for the content.

 The study design, methods and analysis are well conduct and appropriate for the topic.

 The conclusions are relevant in this field.

                                                      

 I think that this article is suitable in this form

I have read this submission. I believe that I have an appropriate level of expertise to confirm that it is of an acceptable scientific standard.

# Peer review: Referee response for version 1

**DOI:** 10.5256/f1000research.9385.r13929

**Published:** 2016-05-23

**Authors:** Hannelore Ehrenreich, Anna-Leena Sirén

**Affiliations:** 1 Department of Clinical Neuroscience, Max Planck Institute of Experimental Medicine, Göttingen, Germany; 2 Department of Neurosurgery, University of Würzburg, Würzburg, Germany

**EPO, TBI and some more to say**

 In their opinion article, *“Is erythropoietin a worthy candidate for traumatic brain injury or are we heading the wrong way?* ***” ***Giovanni Grasso, Concetta Alafaci and Pietro Ghezzi address a very important topic. Specifically, they summarize and comment on recent formally negative clinical trials on the use of recombinant human erythropoietin (EPO) in traumatic brain injury (TBI). More globally, they lay their fingers in the wound of numerous clinical trials on neuroprotection and neuroregeneration in brain diseases that failed in translation from preclinical studies to the patient. The authors discuss briefly properties and encouraging preclinical studies on EPO, and then raise the most critical issues of the human EPO trials in TBI, mainly the late administration of the first dose, the short duration and low frequency of treatment - all essentially dictated by not too well established safety concerns.

 While we fully agree on most of these points, we do not think that the single dose was necessarily too low, as also criticized by the authors. We feel that the dose was likely sufficient but that intravenous rather than subcutaneous dosing should have been used to achieve higher EPO levels in the brain.

 We would like to add a few comments that we feel – after 20 years of own experience in translational work on the brain EPO system – are important to consider.

 We had to painfully learn ourselves the downstream consequences of pharmaceutical companies and overeager regulatories drawing fast conclusions out of too superficially or not at all analyzed data. This triggered an avalanche of destruction regarding our large EPO stroke multicenter trial where severe treatment violations of stroke patients in several centers (totally independent of the study medication) explained the outcome rather than EPO ^1^. In fact, careful subpopulation analysis of all dead patients revealed that several relevant baseline characteristics (i.e. data obtained *before* administration of any study medication) were significantly different between groups, always in disadvantage of the EPO group. Most importantly, upon inclusion (before any study drug application), intent-to-treat non-rtPA receiving EPO patients who died suffered from much severer strokes as compared to placebo patients (NIHSS day 1: 20.4±5.4 versus 13.3±4.9; p=0.003). This highly significant prediction of a worse outcome explains the twofold higher very early death rate in the EPO group ( http://www.epo-study.de/index_eng.html).

 Unfortunately, the premature jumping to conclusions regarding the EPO stroke trial influenced also one of the TBI trials discussed here: The authors write that *“there was concern by the FDA that the initial regimen of 3 daily doses of EPO would impose a greater risk of death. This concern resulted in a modified study design after approximately one-third of the patients had been enrolled in the trial. We did not detect an increased mortality rate with the EPO dose regimen, and the neurological outcome results were more promising than with the subsequent regimen. However, because the original dose regimen was stopped early, the numbers of cases are too small to draw any conclusions”* ^2^.

 Even though safety is undoubtedly an important issue, we have to reduce the incredible arrogance of dismissing any trial that shows ‘just signals’ of benefit. How can we expect more in an initial translational step? Being aware that there are no neuroprotective/neuroregenerative treatments out for conditions as frequent and detrimental as stroke or TBI, it would be time to work hard on any positive signals rather than ‘throw the baby out with the bathwater’.

 And clearly, both TBI trials ^2^^,^^3^ show such signals. Regarding stroke, not only the first EPO trial was obviously promising ^4^, but also the retrospective analysis of patients from Hannover, the most efficiently recruiting center of the second EPO stroke trial ^5^, made the beneficial effect of EPO in stroke again very obvious.

 Getting back to the opinion paper by Grasso and colleagues, longer treatment duration - over many weeks - may ultimately disclose the benefit of EPO also for TBI much stronger. Clinical studies on EPO in chronic brain diseases (schizophrenia, multiple sclerosis, major and bipolar depression) with extended treatment using high dose EPO over many weeks showed consistently advantageous effects on cognition, motor function, and even reduction of brain matter loss. All these findings were in absence of any appreciable side effects ^6^^-^^11^. Of course, in all clinical studies, the quality of patient care including alert follow-up of individual patients at all times is mandatory ^12^.

 Work on EPO indications outside the hematopoietic system has been difficult ever since. Large studies would be needed with adequate funding. Funding agencies do not provide enough money and direct requests for financing to industry. Industry has not been supportive due to expired EPO patents and many EPO biosimilar producers popping up, increasing the risk of ‘off-label-use’. Protection of the extremely lucrative anemia market includes avoiding the risk of additional side effects in new indications.  

 EPO is a potent growth factor, not a miracle drug, and it is not a causal treatment or cure of brain diseases but it may improve their outcome. Even though doping is an unpleasant chapter in itself, it may be seen in the present context as one of the most convincing field studies ever regarding efficiency of EPO. Who would invest huge amounts of money over decades and risk to be caught by controllers and convicted if EPO were not extremely effective?

 Despite all frustrations and disappointments: We have to keep trying to understand how EPO acts in the brain and to ultimately exploit this knowledge for the benefit of our patients. Some novel and surprising insight, lending further support to the use of EPO for neuroprotective and neuroregenerative treatment of brain diseases, comes from recent studies showing that EPO increases the number of neurons and oligodendrocytes in the hippocampus by driving pre-existing precursors to differentiate ^13^, or from work reporting EPO effects on synaptic plasticity ^14^^,^^15^.

We have read this submission. We believe that we have an appropriate level of expertise to confirm that it is of an acceptable scientific standard.

## References

[1] EhrenreichHWeissenbornKPrangeHSchneiderDWeimarCWartenbergKSchellingerPDBohnMBeckerHWegrzynMJähnigPHerrmannMKnauthMBährMHeideWWagnerASchwabSReichmannHSchwendemannGDenglerRKastrupABartelsC: Recombinant human erythropoietin in the treatment of acute ischemic stroke. *Stroke*.2009;40(12) : 10.1161/STROKEAHA.109.564872 e647-56 10.1161/STROKEAHA.109.564872 19834012

[2] RobertsonCSHannayHJYamalJMGopinathSGoodmanJCTilleyBCBaldwinARivera LaraLSaucedo-CrespoHAhmedOSadasivanSPonceLCruz-NavarroJShahinHAisikuIPDoshiPValadkaANeipertLWaguspackJMRubinMLBenoitJSSwankP: Effect of erythropoietin and transfusion threshold on neurological recovery after traumatic brain injury: a randomized clinical trial. *JAMA*.2014;312(1) : 10.1001/jama.2014.6490 36-47 10.1001/jama.2014.6490 25058216PMC4113910

[3] NicholAFrenchCLittleLHaddadSPresneillJArabiYBaileyMCooperDJDuranteauJHuetOMakAMcArthurCPettiläVSkrifvarsMVallanceSVarmaDWillsJBellomoR: Erythropoietin in traumatic brain injury (EPO-TBI): a double-blind randomised controlled trial. *Lancet*.2015;386(10012) : 10.1016/S0140-6736(15)00386-4 2499-506 10.1016/S0140-6736(15)00386-4 26452709

[4] EhrenreichHHasselblattMDembowskiCCepekLLewczukPStiefelMRustenbeckHHBreiterNJacobSKnerlichFBohnMPoserWRütherEKochenMGefellerOGleiterCWesselTCDe RyckMItriLPrangeHCeramiABrinesMSirénAL: Erythropoietin therapy for acute stroke is both safe and beneficial. *Mol Med*.2002;8(8) :495-505 12435860PMC2040012

[5] WorthmannHMartens-LobenhofferJJoumaahMLiNLichtinghagenRHeckerHKielsteinJTEhrenreichHBode-BögerSMWeissenbornK: Asymmetric dimethylarginine in response to recombinant tissue-type plasminogen activator and erythropoietin in acute stroke. *Stroke*.2013;44(8) : 10.1161/STROKEAHA.113.001145 2128-33 10.1161/STROKEAHA.113.001145 23788583

[6] EhrenreichHFischerBNorraCSchellenbergerFStenderNStiefelMSirénALPaulusWNaveKAGoldRBartelsC: Exploring recombinant human erythropoietin in chronic progressive multiple sclerosis. *Brain*.2007;130(Pt 10) : 10.1093/brain/awm203 2577-88 10.1093/brain/awm203 17728357

[7] EhrenreichHHinze-SelchDStawickiSAustCKnolle-VeentjerSWilmsSHeinzGErdagSJahnHDegnerDRitzenMMohrAWagnerMSchneiderUBohnMHuberMCzernikAPollmächerTMaierWSirénALKlosterkötterJFalkaiPRütherEAldenhoffJBKrampeH: Improvement of cognitive functions in chronic schizophrenic patients by recombinant human erythropoietin. *Mol Psychiatry*.2007;12(2) : 10.1038/sj.mp.4001907 206-20 10.1038/sj.mp.4001907 17033631

[8] WüstenbergTBegemannMBartelsCGefellerOStawickiSHinze-SelchDMohrAFalkaiPAldenhoffJBKnauthMNaveKAEhrenreichH: Recombinant human erythropoietin delays loss of gray matter in chronic schizophrenia. *Mol Psychiatry*.2011;16(1) : 10.1038/mp.2010.51 26-36, 1 10.1038/mp.2010.51 20479759

[9] MiskowiakKWEhrenreichHChristensenEMKessingLVVinbergM: Recombinant human erythropoietin to target cognitive dysfunction in bipolar disorder: a double-blind, randomized, placebo-controlled phase 2 trial. *J Clin Psychiatry*.2014;75(12) : 10.4088/JCP.13m08839 1347-55 10.4088/JCP.13m08839 25099079

[10] MiskowiakKWVinbergMChristensenEMBukhJDHarmerCJEhrenreichHKessingLV: Recombinant human erythropoietin for treating treatment-resistant depression: a double-blind, randomized, placebo-controlled phase 2 trial. *Neuropsychopharmacology*.2014;39(6) : 10.1038/npp.2013.335 1399-408 10.1038/npp.2013.335 24322509PMC3988543

[11] MiskowiakKWVinbergMMacoveanuJEhrenreichHKøsterNInksterBPaulsonOBKessingLVSkimmingeASiebnerHR: Effects of Erythropoietin on Hippocampal Volume and Memory in Mood Disorders. *Biol Psychiatry*.2015;78(4) : 10.1016/j.biopsych.2014.12.013 270-7 10.1016/j.biopsych.2014.12.013 25641635

[12] SirénALFasshauerTBartelsCEhrenreichH: Therapeutic potential of erythropoietin and its structural or functional variants in the nervous system. *Neurotherapeutics*.2009;6(1) : 10.1016/j.nurt.2008.10.041 108-27 10.1016/j.nurt.2008.10.041 19110203PMC5084260

[13] HassounaIOttCWüstefeldLOffenNNeherRAMitkovskiMWinklerDSperlingSFriesLGoebbelsSVrejaICHagemeyerNDittrichMRossettiMFKröhnertKHannkeKBoretiusSZeugAHöschenCDandekarTDereENeherERizzoliSONaveKASirénALEhrenreichH: Revisiting adult neurogenesis and the role of erythropoietin for neuronal and oligodendroglial differentiation in the hippocampus. *Mol Psychiatry*.2016; 10.1038/mp.2015.212 10.1038/mp.2015.212 26809838PMC5193535

[14] Almaguer-MelianWMercerón-MartínezDDelgado-OcañaSPavón-FuentesNLedónNBergadoJA: EPO induces changes in synaptic transmission and plasticity in the dentate gyrus of rats. *Synapse*.2016;70(6) : 10.1002/syn.21895 240-52 10.1002/syn.21895 26860222

[15] MengozziMCervelliniIVillaPErbayraktarZGökmenNYilmazOErbayraktarSManohasandraMVan HummelenPVandenabeelePChernajovskyYAnnenkovAGhezziP: Erythropoietin-induced changes in brain gene expression reveal induction of synaptic plasticity genes in experimental stroke. *Proc Natl Acad Sci U S A*.2012;109(24) : 10.1073/pnas.1200554109 9617-22 10.1073/pnas.1200554109 22645329PMC3386063
